# Mammarenavirus Z Protein Myristoylation and Oligomerization Are Not Required for Its Dose-Dependent Inhibitory Effect on vRNP Activity

**DOI:** 10.3390/biochem5020010

**Published:** 2025-04-29

**Authors:** Haydar Witwit, Juan C. de la Torre

**Affiliations:** Department of Immunology and Microbiology, The Scripps Research Institute, La Jolla, CA 92037, USA

**Keywords:** mammarenavirus, multimerization, ribonucleoprotein inhibition, Z matrix protein, myristoylation, N-myristoyltransferases, antiviral, LCMV, LASV, proteasome, glycoprotein, signal peptide

## Abstract

**Background/Objectives:**

N-Myristoyltransferase inhibitors (NMTi) represent a novel antiviral strategy against mammarenaviruses such as Lassa and Junin viruses. The Z matrix protein inhibits viral ribonucleoprotein (vRNP) activity in a dose-dependent manner. Here, we investigated whether Z-mediated vRNP inhibition depends on Z myristoylation or oligomerization.

**Methods:**

We used HEK293T cells transfected with wild-type (WT) or G2A-mutated Z constructs in LCMV minigenome (MG) assays. Cells were treated with the NMTi IMP-1088 and the proteasome inhibitor MG132. Z protein expression, vRNP activity, and VLP production were analyzed by immunofluorescence, western blotting, and colocalization analyses.

**Results:**

IMP-1088 treatment led to proteasome-mediated degradation of Z, reducing its inhibition of vRNP activity, which was restored by MG132. The non-myristoylated Z G2A mutant retained vRNP inhibitory activity but showed impaired oligomerization and budding capacity. These findings demonstrate that Z-mediated vRNP inhibition is independent of myristoylation and oligomerization.

**Conclusions:**

Z myristoylation and oligomerization are not required for its inhibitory vRNP activity. Targeting Z myristoylation with NMTi impairs virus assembly and budding without affecting Z-mediated inhibition of vRNP activity, supporting the development of NMTi as a promising broad-spectrum antiviral strategy against mammarenaviruses.

## 1. Introduction

Several mammarenaviruses, chiefly Lassa virus (LASV) in Western Africa and Junin virus (JUNV) in the Argentine Pampas cause hemorrhagic fever (HF) diseases associated with high morbidity and mortality, posing important public health problems in their endemic regions. In addition, mounting evidence indicates that the globally distributed mammarenavirus lymphocytic choriomeningitis virus (LCMV) is a neglected human pathogen of clinical significance in pediatric and transplantation medicine [1–3]. Current anti-mammarenavirus therapy is limited to an off-label use of ribavirin, for which efficacy remains controversial [4]. Hence, the importance of developing novel therapeutics to combat human pathogenic mammarenaviruses.

Mammarenaviruses are enveloped viruses with a bi-segmented negative-stranded (NS) RNA genome [5]. Each genome segment, L (ca 7.3 kb) and S (ca 3.5 kb), uses an ambisense coding strategy to direct the synthesis of two polypeptides in opposite orientations, separated by a non-coding intergenic region (IGR). The L segment encodes the viral RNA-dependent RNA polymerase (L) and Z matrix protein, whereas the S segment encodes the viral glycoprotein precursor (GPC) and the viral nucleoprotein (NP). GPC is co- and post-translationally processed to produce a stable signal peptide (SSP), and the mature GP1 and GP2 subunits that together with the SSP form the spikes that decorate the virus surface and mediate cell entry via receptor-mediated endocytosis [6–8]. GP1 mediates binding to the cellular receptor and GP2 mediates the pH-dependent fusion event in the late endosome required for the release of vRNP into the cell cytoplasm, where it directs replication and transcription of the viral genome [9,10].

**Figure 1.**
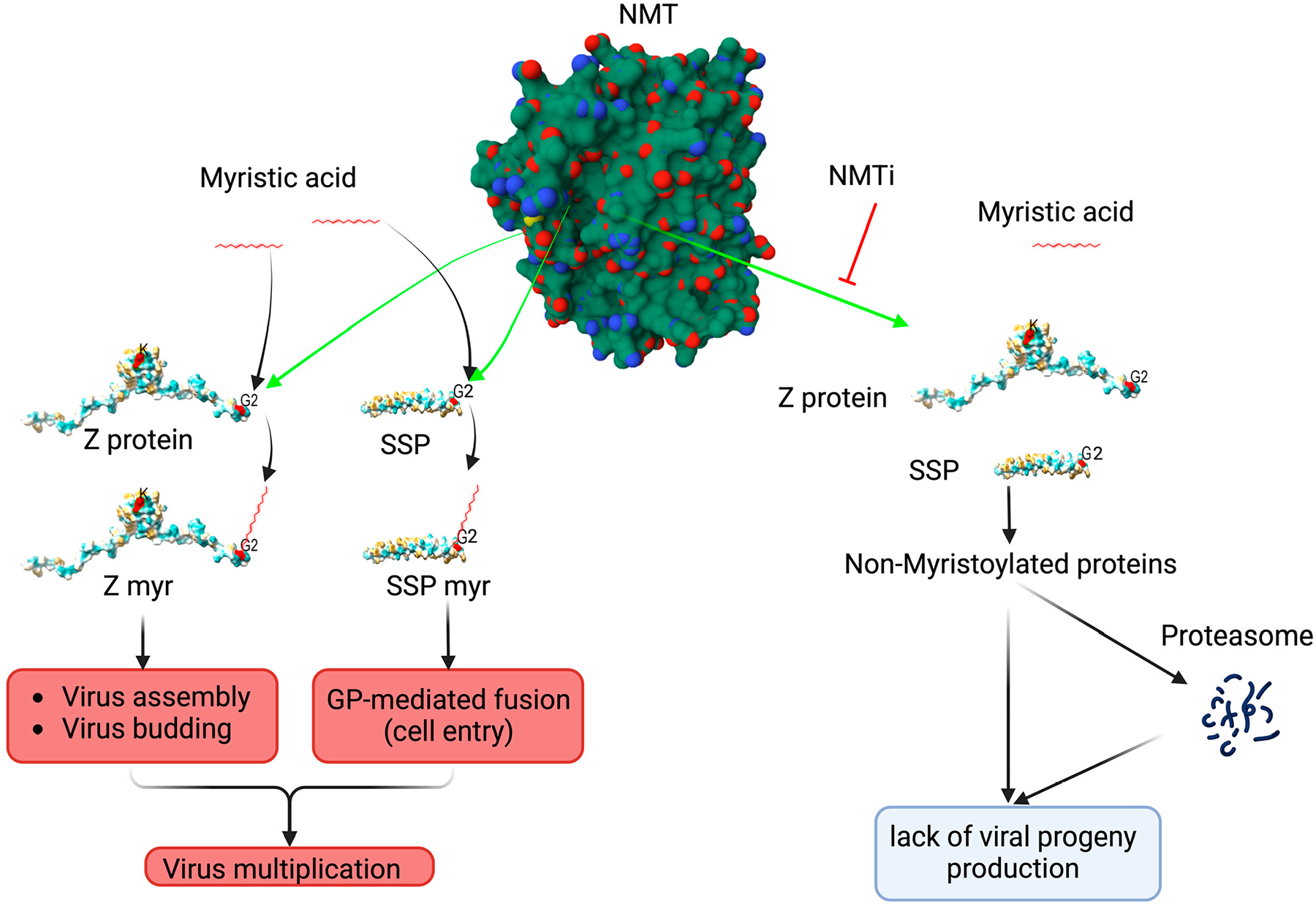
Proposed model of the effect of the NMT inhibitor on mammarenavirus cell entry and budding. NMT isozymes facilitate the addition of myristic acid to glycine (2) of SSP and Z protein, which protect them from proteasome-mediated degradation. Myristoylated SSP interacts with GP2 to facilitate the fusion event in the late endosome required to complete the virus cell entry process, whereas myristoylated Z directs the virus assembly and budding process. Inhibition of SSP and Z myristoylation by NMT inh results in proteasome-mediated degradation of SSP and Z, which results in the inhibition of virus multiplication. Z myr and SSP myr indicate myristoylated Z and SSP, respectively. NMT pdb, 3IWE [25], and LASV Z matrix protein pdb, 2M1S [26], were used to generate the 3D figure using ChimeraX [27].

Early studies have shown that N-myristoylation is required for the role of Z protein in assembly and budding [11,12] and for the role of the SSP in the GP2-mediate fusion event [9]. These findings were based on the use of 2-hydroxy-myristic acid (2-HMA) and 2-HMA analogs as inhibitors of N-myristoyltransferase 1 (NMT1) and 2 (NMT2) responsible for catalyzing N-myristoylation in mammalian cells [13]. However, recent studies have demonstrated that 2-HMA acts off-target and does not inhibit N-myristoylation within a concentration range consistent with activity on NMT [14]. Therefore, we revisited the contribution of N-myristoylation in mammarenavirus infection using the validated on-target specific pan-NMTi (DDD85646 and IMP-1088) [15]. We found that DDD85646 and IMP-1088 exhibit very potent antiviral activity against LCMV and LASV in cultured cells. Cell-based assays probing different steps of the LCMV life cycle revealed that NMTi exerted its anti-LCMV activity by interfering with Z budding activity and GP2-mediated fusion [16] (Figure 1). Our findings support the use of NMTi as a novel host-targeted antiviral strategy to combat LASV and other human pathogenic mammarenaviruses. Moreover, NMTi can be incorporated into combination therapies with direct-acting antivirals. By targeting a host cell factor, NMTi can impose a high genetic barrier against the selection of drug-resistant variants, a common problem with direct-acting antiviral drugs. While Z dose-dependent suppression of vRNP activity is well documented [17–19], there is limited knowledge about the underlying mechanism [20–22]. Z protein has been shown to exhibit different degrees of oligomerization [16,23,24], whose biological implications remain poorly understood. Here, we present evidence that Z oligomerization might be required for its budding activity, but not for its ability to inhibit vRNP activity.

## 2. Materials and Methods

### 2.1. Cell Line and Compounds

Human embryonic kidney cells stably expressing T antigen using the SV40 promoter (HEK293T) (ATCC CRL-3216) were maintained in Dulbecco’s Modified Eagle’s Medium (DMEM) supplemented with 10% fetal bovine serum, 1 mM penicillin and streptomycin, and 1 mM glutamine. Cells were incubated at 37C and supplemented with 5% CO_2_. NMTi IMP-1088, (5-[3,4-difluoro-2-[2-(1,3,5-trimethyl-1H-pyrazol-4-yl)ethoxy]phenyl]-N,N,1-trimethyl-1H-indazole-3-methanamine, Cat No. 25366-1) was purchased from Cayman Chemical (Ann Arbor, MI, USA), dissolved in methyl acetate at 11 mM, and kept in aliquots at −20. MG132 (MFG No. M7449) was purchased from Sigma-Aldrich (St. Louis, MO, USA).

### 2.2. Plasmids

The ORF of the LCMV Z gene was cloned into pCAGGS and labeled with an HA-tag, referred to here as Z-WT-HA, as described previously [16]. T7 polymerase in the pCAGGS system and the S segment of LCMV, flanked by a T7 promoter at the amino terminus, was used to express GFP. Both LCMV L polymerase and NP are expressed in pCAGGS systems.

### 2.3. Minigenome Assay

HEK293 cells were seeded at 2 × 10^5^ per well in poly-L-lysine-treated M12-well plates. The next day, cells were transfected with plasmids expressing LCMV L and NP proteins, the minimal transacting viral factors required for replication and transcription of the viral genome. Also included was a plasmid directing intracellular synthesis of an LCMV minigenome directing expression of GFP (MG-GFP), together with a plasmid expressing the T7 RNA polymerase to launch primary synthesis of the MG-GFP, and incremental amounts of a plasmid expressing LCMV-Z tagged with HA (Z-WT-HA). At 18 h post-transfection, cells were treated with the indicated compounds, and at 24 h post-treatment, cells were fixed with 4% paraformaldehyde (PFA), and stained with anti-HA, followed by a secondary fluorescence antibody to identify cells expressing the Z protein. The activity of the LCMV MG-GFP was assessed based on expression levels of GFP. Images were acquired at 4X magnification (Keyence Corporation, Itasca, IL, USA).

### 2.4. VLP Assay

Viral-like particle (VLP) production was performed as previously described [11]. Briefly, HEK293T cells were transfected with pC_E as the control, Z-WT or the G2A mutant, and cell culture supernatant (CCS) was collected at 48 or 72 h post-transfection (h pt). CCS were clarified by centrifugation (5 min, 5000 RPM, 4 °C). VLPs in clarified CCS were collected by ultracentrifugation (100,000× *g*, 2 h, 4 °C) through a 20% sucrose cushion in 50 mM Tris-HCl pH 7.5, 62.5 mM EDTA, 1% NP-40, 0.4% Na deoxycholoate. The pellet containing VLPs was resuspended in 60 μL of PBS and 20 μL of 4X Laemmli buffer was added.

### 2.5. Immunoblotting

Protein concentration in samples was determined by BCA assay (Pierce^™^ BCA Protein Assay Kits, Cat# 23227, ThermoFisher, Waltham, MA, USA). Samples (12 μg) were heated for 5 min at 95 °C and separated by SDS-PAGE using a stain-free gel (Bio-Rad, Hercules, CA, USA). Total protein (TP) was detected after the activation of the stain-free gel for one minute. Proteins were transferred to a low-fluorescence PVDF membrane (Bio-Rad, Hercules, CA, USA), and EveryBlot blocking buffer (Bio-Rad, Hercules, CA, USA) was used to block nonspecific antibody binding. The membrane was immunoreacted with an anti-HA antibody (Genscript, Piscataway, NJ, USA) overnight at 4 °C. After three washes, the membrane was immunoreacted with a horseradish peroxidase (HRP) conjugated with anti-mouse antibody. Primary and secondary antibodies were diluted in a OneBlock western-CL blocking buffer (Genesee Scientific, San Diego, CA, USA). Protein bands were visualized using a chemiluminescent substrate (ThermoFisher Scientific).

### 2.6. Immunofluorescence Imaging

Images were acquired at 4X magnification using a BZ-X710 Keyence fluorescence microscope. The quantification and depiction of Z and GFP were carried out using the BIOP JaCoP plugin, a colocalization quantification software, in FIJI imagej software (1.54f). The Otsu threshold setting was selected to quantify the degree of colocalization. The BIOP JaCoP generates, based on a threshold setting, an automated figure that on the left side displays unmasked (top) and masked (bottom) panels that represent the Pearson correlation coefficient (*X* axis) and probability (*Y* axis) plots, and on the right side displays a scatter plot representing the GFP (green, *Y* axis) and Z-HA (red, *X* axis) signals. The rationale to use the BIOP Jacob plug is to quantify colocalization of GFP (a surrogate of the MG activity) and the AlexaFLuor 568 red signal (a surrogate of Z matrix protein expression).

## 3. Results

### 3.1. Effect of NMTi on Z-Mediated Inhibition of vRNP Activity

**Figure 2.**
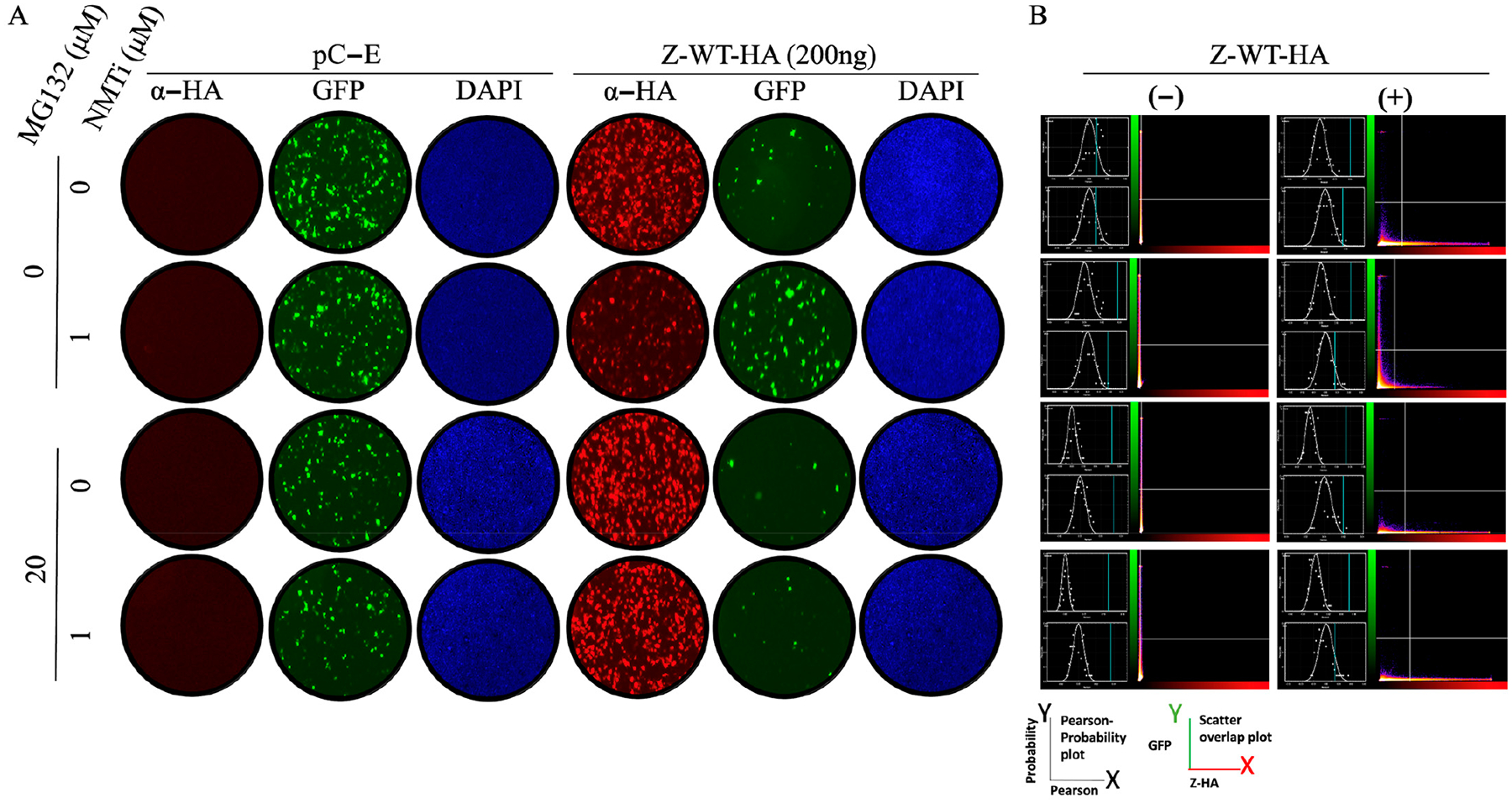
Effect of treatments with the NMTi (IMP-1088) and proteasome (MG132) inhibitors on the activity of the LCMV MG in the presence of LCMV WT Z protein. HEK293T cells were seeded at 2 × 10^5^ cells per well in poly-L-lysine-treated M12-well plates. The next day, cells were transfected with pCAGGS plasmids expressing LCMV L and NP proteins, as well as T7RP, and a plasmid expressing the LCMV T7MG-GFP, together with 200 ng of a pCAGGS empty (pC-E) plasmid or expressing LCMV-Z -WT-HA. At 18 h post-transfection, cells were treated with the indicated compounds, and at 24 h post-treatment, cells were fixed with 4% PFA. Z-WT-HA expression was detected by IF using a rabitt polyclonal antibody to α-HA as primary antibody and an anti-mouse goat polyclonal antibody conjugated to Alexafluor 568 as secondary antibody. GFP was detected directly by epifluorescence. (**A**). The activity of LCMV MG-GFP was assessed based on expression levels of GFP. (**B**). The left side of each panel displays unmasked (**top**) and masked (**bottom**) panels that represent the Pearson correlation coefficient (*X* axis) and probability (*Y* axis) plots, and on the right side displays a scatter plot representing the GFP (green, *Y* axis) and Z-HA (red, *X* axis) signals. The BIOP Jacob plug quantified colocalization of GFP (a surrogate of the MG activity) and AlexaFLuor 568 red signal (a surrogate of Z matrix protein expression).

The Z protein has been shown to inhibit, in a dose-dependent manner, the activity of the mammarenavirus vRNP, which is responsible for directing the replication and transcription of the viral genome, in cell-based minigenome (MG) assays. We have documented that treatment with NMTi targets Z for degradation via the proteasome pathway [16]. We therefore predicted that the Z inhibitory effect on MG activity would be reduced in the presence of the pan-NMTi IMP-1088, and that treatment with the proteasome inhibitor MG132 would restore, in the presence of IMP-1088, the Z inhibitory effect on MG activity. Using a cell-based LCMV minigenome (MG) system we found that treatment with the NMTi IMP-1088 counteracts the Z-mediated inhibitory effect on MG activity (Figure 2). Treatment with the proteasome inhibitor MG132 restored expression levels of Z to those observed in the absence of IMP-1088, which resulted in the corresponding inhibitory effect on the activity of the LCMV MG (Figure 2).

### 3.2. Role of N-Myristoylation on Z-Mediated Inhibition of vRNP Activity

**Figure 3.**
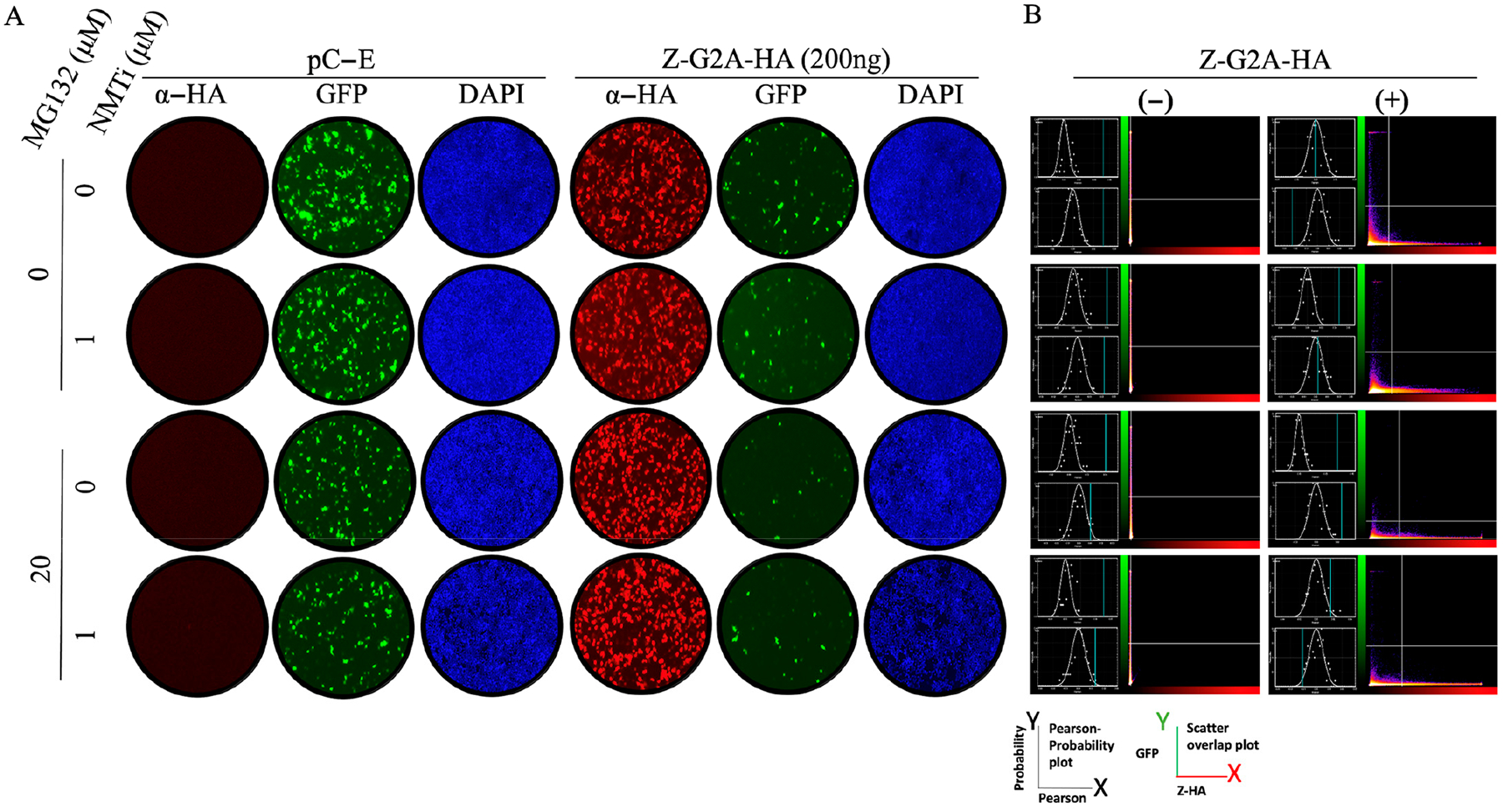
Effect of treatments with the NMT (IMP-1088) and proteasome (MG132) inhibitors on the activity of the LCMV MG in the presence of the LCMV Z G2A mutant. HEK293T cells were seeded at 2 × 10^5^ cells per well in poly-L-lysine-treated M12 well plates. The next day, cells were transfected with pCAGGS plasmids expressing LCMV L and NP proteins, as well as T7RP, and a plasmid expressing the LCMV T7 MG-GFP, together with 200 ng of a pCAGGS empty (pC-E) plasmid or a plasmid expressing LCMV-Z-G2A-HA. At 18 h post-transfection, cells were treated with the indicated compounds, and at 24 h post-treatment, cells were fixed with 4% PFA. Z-G2A-HA expression was detected by IF using a rabitt polyclonal antibody to α-HA as a primary antibody and an anti-mouse goat polyclonal antibody conjugated to Alexafluor 568 as a secondary antibody. GFP was detected directly by epifluorescence. (**A**). The activity of LCMV MG-GFP was assessed based on the expression levels of GFP. (**B**). The left side of each panel displays unmasked (**top**) and masked (**bottom**) panels that represent the Pearson correlation coefficient (*X* axis) and probability (*Y* axis) plots, and the right side displays a scatter plot representing the GFP (green, *Y* axis) and Z-G2A-HA (red, *X* axis) signals. The BIOP Jacob plug quantified colocalization of GFP (a surrogate of the MG activity) and the AlexaFLuor 568 red signal (a surrogate of Z matrix protein expression).

Treatment with NMTi targeted Z protein for degradation, which prevented us from assessing whether myristoylation was required for Z-mediated inhibition of vRNP activity. To address this question, we used the Z (G2A) mutant that cannot undergo N-terminal myristoylation. As predicted, treatment with IMP-1088 did not significantly affect expression levels of Z (G2A) (Figure 3). Notably, Z (G2A) inhibited MG-directed GFP expression with a similar efficiency to Z WT (Figure 3), indicating that myristoylation is not required for Z-mediated inhibition of vRNP activity.

### 3.3. Role of Z Oligomerization on Z-Mediated Inhibition of vRNP Activity and Z Budding Activity

**Figure 4.**
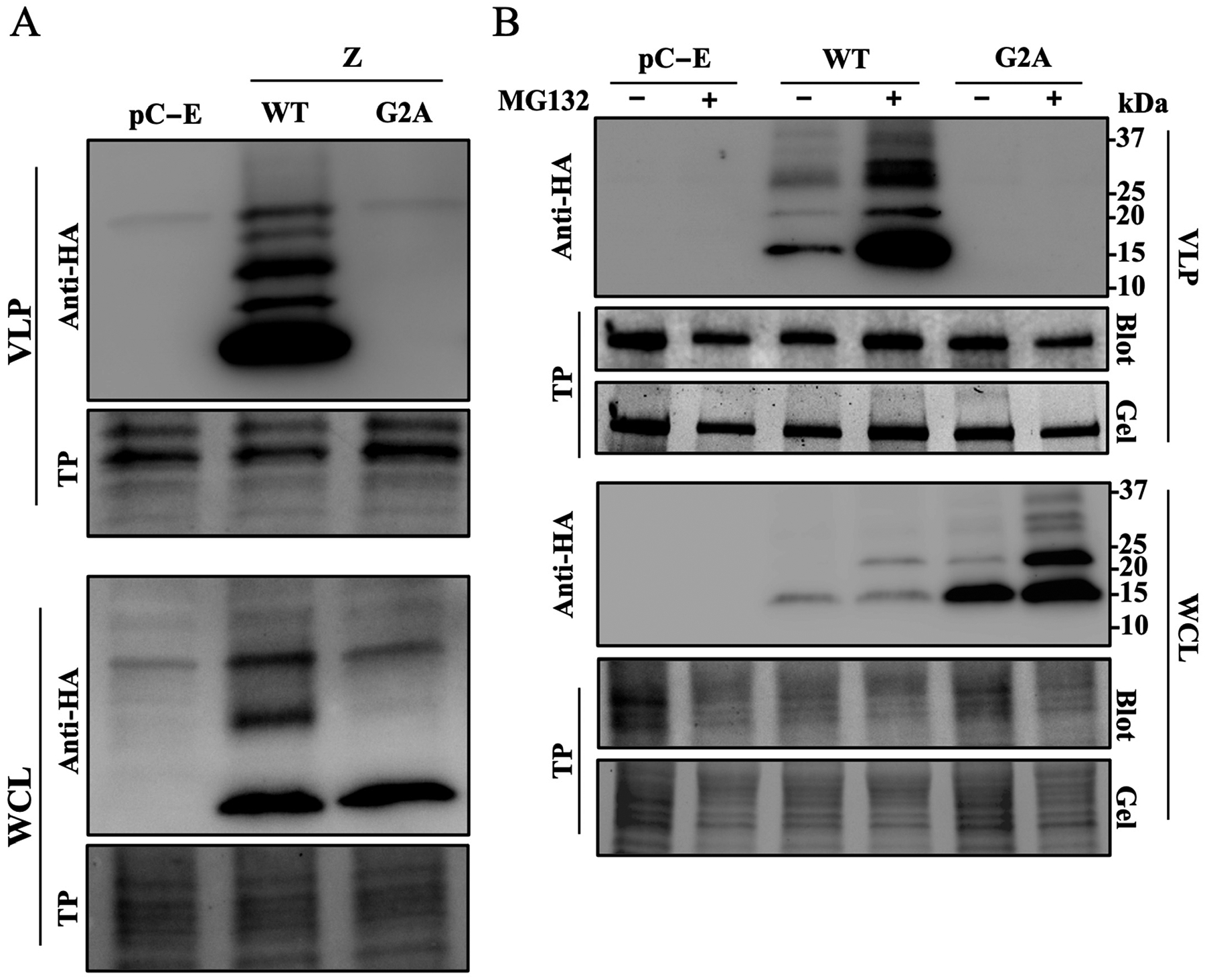
Oligomerization and cell egress of Z protein. (**A**). HEK293T cells were seeded at 1 × 10^6^ cells per well in poly-L-lysine-treated M6-well plates. The next day, cells were transfected with plasmids expressing LCMV Z WT or G2A mutant proteins tagged with an HA tag or empty pCAGGS (pC-E). At 72 h post-transfection, supernatant and protein cell lysate were collected to extract VLP or whole-cell lysate (WCL) protein, respectively. (**B**). HEK293T cells were seeded at 1 × 10^6^ cells per well in poly-L-lysine-treated M6-well plates. The next day, cells were transfected with plasmids expressing LCMV Z WT or G2A mutant proteins tagged with an HA tag or pC-E. The next day, media were aspirated and replaced with media containing MG132 (10 μg/mL) or a vehicle control. At 48 h post-transfection, supernatants were collected and whole cell lysate (WCL) was prepared. VLPs were collected by ultracentrifugation. Levels of Z in VLP and WCL samples were determined by Western blotting using an antibody to HA. Membranes were imaged for total protein (TP) prior to probing with an anti-HA antibody.

We have documented the detection of different oligomer species of Z protein in lysates of Z transfected cells using Western blotting [16]. We detected these Z oligomeric species in VLPs produced in cells expressing WT, but not in cells expressing mutant G2A or Z protein (Figure 4) mutant. In addition, treatment with the proteasome inhibitor MG132 rescued high expression levels of dimer and oligomers of G2A mutant form of Z in WCL, but these Z(G2A) species lacked detectable levels of budding activity as determined by their absence in the corresponding VLP samples (Figure 4B). These findings are consistent with published results showing the lack of Z oligomeric species in lysates and Z-containing VLP in cell culture supernatants of cells transfected with Z-G2A-HA [11,16]. We observed four Z species in VLP produced in cells transfected with Z-WT-HA, with monomeric Z being the most abundant Z species. Inhibition of Z myristoylation using NMTi or mutation G2A results in the degradation of Z oligomeric species [16], which complicates discerning the relative contributions of myristoylation and oligomerization to Z budding activity.

## 4. Discussion

The mammarenavirus Z matrix protein plays critical roles in the assembly and budding of matured infectious particles, processes that require Z myristoylation and oligomerization [11,24,28]. In addition, as with the matrix protein of other negative strand RNA viruses [29], Z protein has been shown to exhibit a dose-dependent inhibitory effect on mammarenavirus vRNP activity [18,19,30]. Biochemical and structural studies have indicated that Z can lock a polymerase-promoter complex [31] and induce conformational changes in L catalytic domains [20], which may account for Z-mediated inhibition of vRNP-directed synthesis of viral RNA. These studies, however, did not address the question of whether myristoylation and oligomerization are required for Z-mediated inhibition of vRNP activity in infected cells.

Here, we have documented that NMTi potent antiviral activity against LCMV and other mammarenaviruses correlated with proteasome-mediated degradation of non-myristoylated Z protein, which disrupted virus particle assembly and budding, resulting in reduced production of infectious progeny and restricted virus propagation [16] without inhibition of vRNP activity. Treatment with the proteasome inhibitor MG132 in the presence of NMTi restored Z-mediated inhibition of vRNP activity (Figure 2), and the Z G2A mutant inhibited the activity of the LCMV MG with similar efficiency to Z WT (Figure 3). These findings indicate that Z myristoylation is not required for its inhibitory effect on vRNP activity. We also observed that Z G2A exhibited significantly reduced levels of oligomerization compared to Z WT (Figure 4), suggesting that oligomerization is not required for the Z inhibitory effect on vRNP activity.

**Figure 5.**
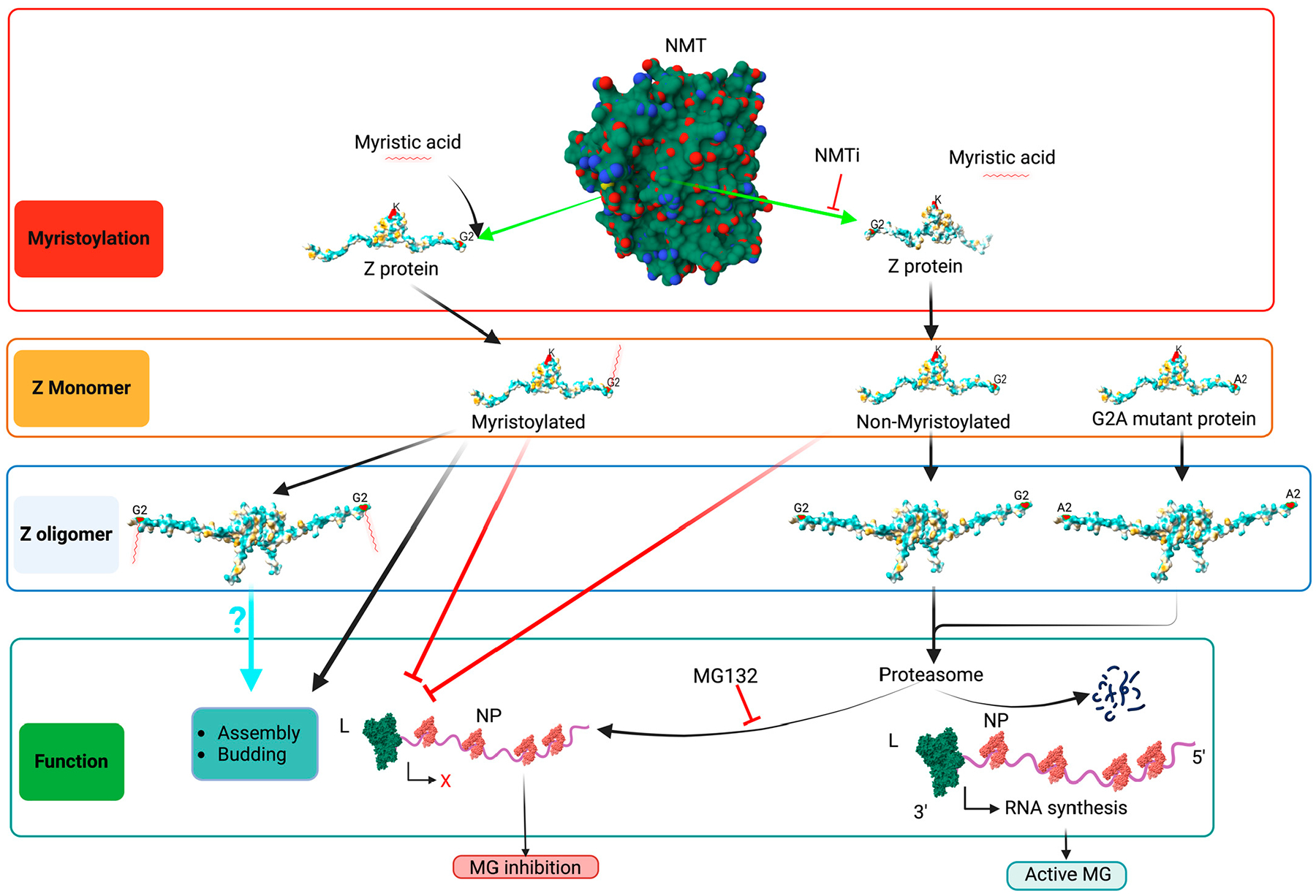
Proposed model of the effect of the NMT inh (IMP-1088) on the activity of the LCMV MG, budding, assembly and infectivity. NMT promotes Z myristoylation at G2, which prevents the targeting of Z for degradation by the proteasome machinery, thus allowing for the Z dose-dependent inhibitory effect on viral RNA synthesis mediated by vRNP and the consequent inhibition of MG-directed GFP expression. Inhibition of Z myristoylation by IMP-1088 results in the proteasome-mediated degradation of Z, which will relieve the Z inhibitory effect on the activity of the LCMV MG (right side of the graph). Treatment with the proteasome inhibitor MG132 prevents IMP-1088-induced Z protein degradation, thus resulting in LCMV MG inhibition. L polymerase (green), NP (red) and RNA (purple) are indicated. A red X indicates cessation activity, and a black angled arrow indicates RNA synthesis. Monomers of Z protein are sufficient to inhibit vRNP. A lack of Z myristoylation results in the degradation of Z oligomers, which complicates the assessment of the role of Z oligomers in virus assembly, budding and infectivity. NMT pdb, 3IWE [25], and LASV Z matrix protein pdb, 2M1S [26] were used to generate the 3D figure using ChimeraX [27].

We have shown that dimers are the most abundant oligomer forms of Z in lysates from LCMV-infected or Z-transfected cells, and that these Z homodimers are efficiently targeted for degradation in the presence of NMTi, questioning the conclusion that Z homo-oligomerization is required for Z accumulation at the plasma membrane [24]. Our results also question whether G2 residue is required for oligomerization [24] because treatment with proteasome inhibitor MG132 resulted in similar expression levels of dimers of Z WT and Z G2A [16]. We propose that myristoylation at G2 prevents the targeting of Z for degradation, thus allowing the Z dose-dependent inhibitory effect on viral RNA synthesis mediated by the vRNP. Inhibition of Z myristoylation with NMTi results in proteasome-mediated degradation of Z, thus preventing the Z inhibitory effect on vRNP activity, which can be restored by treatment with the proteasome inhibitor MG132 (Figure 5). We observed a prominent Z species of ~33 kDa, likely a trimer of Z, in cells transfected with Z WT that was absent in cells transfected with the G2A Z mutant and in the presence of MG132. The biological role of this Z species remains to be determined [16]. We also showed that oligomers of the Z-G2A mutant lacked budding activity, compared to Z WT, and were not detected in VLPs, despite being expressed at high levels in WCL in the presence of MG132. These findings further support the notion that N-myristoylation is required for Z budding activity. It remains to be determined whether other protein lipidation modifications such as S-palmitoylation can substitute for N-myristoylation and facilitate Z association to membranes and budding.

The use of NMTi to target host-cell lipidation processes opens the door to a new class of broad-spectrum anti-mammarenavirus therapy. Moreover, NMTi could be also incorporated into combination therapy strategies with direct-acting antivirals, an approach expected to impose a significant genetic barrier to the emergence of drug-resistant viruses, and to facilitate drug formulations with reduced toxicity [32]. Notably, the small molecule NMTi PCLX-001 has been shown to be safe and is well tolerated by humans [33,34], supporting the interest in exploring the repurposing of NMTi to treat infections by human pathogenic mammarenaviruses. NMTi inhibitors have been shown to inhibit other viruses with myristoylated proteins, including picornaviruses [15] and vaccinia virus [32,35]. The use of specific pan-NMTi as a broad-spectrum antiviral strategy against viruses with myristoylated proteins warrants further investigation.

## Data Availability

Data is contained within the article.

## References

[1] Yadav K; Mathur G; Ford B; Miller R; Group CW A Case Cluster of Lymphocytic Choriomeningitis Virus Transmitted Via Organ Transplantation.: Abstract# D2381. Transplantation 2014, 98, 768.

[2] Sayyad LE; Smith KL; Sadigh KS; Cossaboom CM; Choi MJ; Whitmer S; Cannon D; Krapiunaya I; Morales-Betoulle M; Annambhotla P; Severe Non–Donor-Derived Lymphocytic Choriomeningitis Virus Infection in 2 Solid Organ Transplant Recipients. Open Forum Infect. Dis 2025, 12, ofaf002. 10.1093/ofid/ofaf002 PMC11783784 39896983

[3] Schafer IJ; Miller R; Ströher U; Knust B; Nichol ST; Rollin PE; Centers for Disease Control and Prevention (CDC). Notes from the Field: A Cluster of Lymphocytic Choriomeningitis Virus Infections Transmitted Through Organ Transplantation—Iowa, 2013. MMWR Morb. Mortal. Wkly. Rep 2014, 63, 249. 24647402 PMC4584636

[4] Carrillo-Bustamante P; Nguyen THT; Oestereich L; Günther S; Guedj J; Graw F Determining Ribavirin’s Mechanism of Action against Lassa Virus Infection. Sci. Rep 2017, 7, 11693. 28916737 10.1038/s41598-017-10198-0 PMC5601963

[5] Radoshitzky SR; Buchmeier M; de la Torre JC Emerging Viruses: Arenaviridae. In Fields Virology, 7th ed.; Knipe D, Howley P, Whelan S, Eds.; Wolters Kluwer: Philadelphia, PA, USA, 2021; Volume I, ISBN 978-1-975112-54-7.

[6] Kunz S; Edelmann KH; de la Torre J-C; Gorney R; Oldstone MBA Mechanisms for Lymphocytic Choriomeningitis Virus Glycoprotein Cleavage, Transport, and Incorporation into Virions. Virology 2003, 314, 168–178. 14517070 10.1016/s0042-6822(03)00421-5

[7] Rojek JM; Lee AM; Nguyen N; Spiropoulou CF; Kunz S Site 1 Protease Is Required for Proteolytic Processing of the Glycoproteins of the South American Hemorrhagic Fever Viruses Junin, Machupo, and Guanarito. J. Virol 2008, 82, 6045–6051. 18400865 10.1128/JVI.02392-07 PMC2395157

[8] Fedeli C; Moreno H; Kunz S Novel Insights into Cell Entry of Emerging Human Pathogenic Arenaviruses. J. Mol. Biol 2018, 430, 1839–1852. 29705070 10.1016/j.jmb.2018.04.026

[9] York J; Nunberg JH Role of the Stable Signal Peptide of Junín Arenavirus Envelope Glycoprotein in pH-Dependent Membrane Fusion. J. Virol 2006, 80, 7775–7780. 16840359 10.1128/JVI.00642-06 PMC1563716

[10] York J; Nunberg JH Myristoylation of the Arenavirus Envelope Glycoprotein Stable Signal Peptide Is Critical for Membrane Fusion but Dispensable for Virion Morphogenesis. J. Virol 2016, 90, 8341–8350. 27412594 10.1128/JVI.01124-16 PMC5008094

[11] Perez M; Greenwald DL; de La Torre JC Myristoylation of the RING Finger Z Protein Is Essential for Arenavirus Budding. J. Virol 2004, 78, 11443–11448. 15452271 10.1128/JVI.78.20.11443-11448.2004 PMC521847

[12] Capul AA; Perez M; Burke E; Kunz S; Buchmeier MJ; de la Torre JC Arenavirus Z-Glycoprotein Association Requires Z Myristoylation but Not Functional RING or Late Domains. J. Virol 2007, 81, 9451–9460. 17581989 10.1128/JVI.00499-07 PMC1951451

[13] Cordo SM; Candurra NA; Damonte EB Myristic Acid Analogs Are Inhibitors of Junin Virus Replication. Microbes Infect. 1999, 1, 609–614. 10611737 10.1016/s1286-4579(99)80060-4

[14] Kallemeijn WW; Lueg GA; Faronato M; Hadavizadeh K; Grocin AG; Song O-R; Howell M; Calado DP; Tate EW Validation and Invalidation of Chemical Probes for the Human N-Myristoyltransferases. Cell Chem. Biol 2019, 26, 892–900.e4. 31006618 10.1016/j.chembiol.2019.03.006 PMC6593224

[15] Ramljak IC; Stanger J; Real-Hohn A; Dreier D; Wimmer L; Redlberger-Fritz M; Fischl W; Klingel K; Mihovilovic MD; Blaas D; Cellular N-Myristoyltransferases Play a Crucial Picornavirus Genus-Specific Role in Viral Assembly, Virion Maturation, and Infectivity. PLoS Pathog. 2018, 14, e1007203. 30080883 10.1371/journal.ppat.1007203 PMC6089459

[16] Witwit H; Betancourt CA; Cubitt B; Khafaji R; Kowalski H; Jackson N; Ye C; Martinez-Sobrido L; de la Torre JC Cellular N-Myristoyl Transferases Are Required for Mammarenavirus Multiplication. Viruses 2024, 16, 1362. 39339839 10.3390/v16091362 PMC11436053

[17] Cornu TI; de la Torre JC Characterization of the Arenavirus RING Finger Z Protein Regions Required for Z-Mediated Inhibition of Viral RNA Synthesis. J. Virol 2002, 76, 6678–6688. 12050381 10.1128/JVI.76.13.6678-6688.2002 PMC136245

[18] Jácamo R; López N; Wilda M; Franze-Fernández MT Tacaribe Virus Z Protein Interacts with the L Polymerase Protein To Inhibit Viral RNA Synthesis. J. Virol 2003, 77, 10383–10393. 12970423 10.1128/JVI.77.19.10383-10393.2003 PMC228501

[19] López N; Jácamo R; Franze-Fernández MT Transcription and RNA Replication of Tacaribe Virus Genome and Antigenome Analogs Require N and L Proteins: Z Protein Is an Inhibitor of These Processes. J. Virol 2001, 75, 12241–12251. 11711615 10.1128/JVI.75.24.12241-12251.2001 PMC116121

[20] Liu L; Wang P; Liu A; Zhang L; Yan L; Guo Y; Xiao G; Rao Z; Lou Z Structure Basis for Allosteric Regulation of Lymphocytic Choriomeningitis Virus Polymerase Function by Z Matrix Protein. Protein Cell 2023, 14, 703–707. 37038286 10.1093/procel/pwad018 PMC10501185

[21] Cornu TI; de la Torre JC RING Finger Z Protein of Lymphocytic Choriomeningitis Virus (LCMV) Inhibits Transcription and RNA Replication of an LCMV S-Segment Minigenome. J. Virol 2001, 75, 9415–9426. 11533204 10.1128/JVI.75.19.9415-9426.2001 PMC114509

[22] Iwasaki M; de la Torre JC A Highly Conserved Leucine in Mammarenavirus Matrix Z Protein Is Required for Z Interaction with the Virus L Polymerase and Z Stability in Cells Harboring an Active Viral Ribonucleoprotein. J. Virol 2018, 92, e02256–17. 29593035 10.1128/JVI.02256-17 PMC5952140

[23] Hastie KM; Zandonatti M; Liu T; Li S; Woods VL; Saphire EO Crystal Structure of the Oligomeric Form of Lassa Virus Matrix Protein Z. J. Virol 2016, 90, 4556–4562. 26912609 10.1128/JVI.02896-15 PMC4836352

[24] Loureiro ME; Wilda M; Levingston Macleod JM; D’Antuono A; Foscaldi S; Buslje CM; Lopez N Molecular Determinants of Arenavirus Z Protein Homo-Oligomerization and L Polymerase Binding. J. Virol 2011, 85, 12304–12314. 21957305 10.1128/JVI.05691-11 PMC3209362

[25] Qiu W; Hutchinson A; Wernimont A; Lin Y-H; Kania A; Ravichandran M; Kozieradzki I; Cossar D; Schapira M; Arrowsmith CH; Crystal Structure of Human Type-I N-Myristoyltransferase with Bound Myristoyl-CoA and Inhibitor DDD85646. 2010, To be published.

[26] Volpon L; Osborne MJ; Capul AA; de la Torre JC; Borden KLB Structural Characterization of the Z RING-eIF4E Complex Reveals a Distinct Mode of Control for eIF4E. Proc. Natl. Acad. Sci. USA 2010, 107, 5441–5446. 20212144 10.1073/pnas.0909877107 PMC2851782

[27] Meng EC; Goddard TD; Pettersen EF; Couch GS; Pearson ZJ; Morris JH; Ferrin TE UCSF ChimeraX: Tools for Structure Building and Analysis. Protein Sci. 2023, 32, e4792. 37774136 10.1002/pro.4792 PMC10588335

[28] Perez M; Craven RC; De La Torre JC The Small RING Finger Protein Z Drives Arenavirus Budding: Implications for Antiviral Strategies. Proc. Natl. Acad. Sci. USA 2003, 100, 12978–12983. 14563923 10.1073/pnas.2133782100 PMC240730

[29] Baudin F; Petit I; Weissenhorn W; Ruigrok RW In Vitro Dissection of the Membrane and RNP Binding Activities of Influenza Virus M1 Protein. Virology 2001, 281, 102–108. 11222100 10.1006/viro.2000.0804

[30] Cornu TI; Feldmann H; de la Torre JC Cells Expressing the RING Finger Z Protein Are Resistant to Arenavirus Infection. J. Virol 2004, 78, 2979–2983. 14990716 10.1128/JVI.78.6.2979-2983.2004 PMC353761

[31] Kranzusch PJ; Whelan SPJ Arenavirus Z Protein Controls Viral RNA Synthesis by Locking a Polymerase–Promoter Complex. Proc. Natl. Acad. Sci. USA 2011, 108, 19743–19748. 22106304 10.1073/pnas.1112742108 PMC3241799

[32] Witwit H; Cubitt B; Khafaji R; Castro EM; Goicoechea M; Lorenzo MM; Blasco R; Martinez-Sobrido L; de la Torre JC Repurposing Drugs for Synergistic Combination Therapies to Counteract Monkeypox Virus Tecovirimat Resistance. Viruses 2025, 17, 92. 39861882 10.3390/v17010092 PMC11769280

[33] Sangha R; Davies NM; Namdar A; Chu M; Spratlin J; Beauchamp E; Berthiaume LG; Mackey JR Novel, First-in-Human, Oral PCLX-001 Treatment in a Patient with Relapsed Diffuse Large B-Cell Lymphoma. Curr. Oncol 2022, 29, 1939–1946. 35323358 10.3390/curroncol29030158 PMC8947478

[34] Sangha RS; Jamal R; Spratlin JL; Kuruvilla J; Sehn LH; Weickert M; Berthiaume LG; Mackey JR A First-in-Human, Open-Label, Phase I Trial of Daily Oral PCLX-001, an NMT Inhibitor, in Patients with Relapsed/Refractory B-Cell Lymphomas and Advanced Solid Tumors. JCO 2023, 41, e15094. 10.1007/s10637-024-01448-w PMC11327210 38837078

[35] Priyamvada L; Kallemeijn WW; Faronato M; Wilkins K; Goldsmith CS; Cotter CA; Ojeda S; Solari R; Moss B; Tate EW; Inhibition of Vaccinia Virus L1 N-Myristoylation by the Host N-Myristoyltransferase Inhibitor IMP-1088 Generates Non-Infectious Virions Defective in Cell Entry. PLoS Pathog. 2022, 18, e1010662. 36215331 10.1371/journal.ppat.1010662 PMC9584500

